# SPOAN syndrome: a novel mutation and new ocular findings; a case report

**DOI:** 10.1186/s12883-021-02051-9

**Published:** 2021-01-15

**Authors:** Fatemeh Bazvand, Mohammad Keramatipour, Hamid Riazi-Esfahani, Alireza Mahmoudi

**Affiliations:** 1 Farabi eye hospital, Eye research center, Tehran University of Medical Science, Farabi Eye Hospital, Qazvin square, South Kargar Street, Tehran, Iran; 2 Department of Medical Genetics, Faculty of Medicine, Tehran University of Medical Sciences, Tehran, Iran

**Keywords:** SPOAN syndrome, Retinopathy, Electroretinography

## Abstract

**Background:**

To report a novel mutation and new clinical findings in a case with SPOAN syndrome (spastic paraplegia, optic atrophy, neuropathy).

**Case presentation:**

Clinical examination, genetic testing and electroretinography were used to study a 2-year-old child who was referred to our clinic with no visual attention and documented SPOAN syndrome. Fundoscopy revealed optic atrophy, diffuse retinal pigment mottling, severe vascular attenuation, and completely non-vascularized peripheral retina in both eyes. Full-field electroretinogram (ERG) revealed flat responses.

**Conclusions:**

Severe retinopathy and flat full-field ERG responses can occur in SPOAN syndrome.

## Background

SPOAN syndrome, an autosomal recessive neurodegenerative disorder, was reported for the first time in 2005 in a large consanguineous family in Brazil [1]. Clinical characteristics include spastic paraplegia, optic atrophy, and neuropathy (SPOAN syndrome, OMIM #609,541). The disease is characterized by the onset of progressive spastic paraplegia in infancy and progressive motor and sensory axonal neuropathy in later childhood, leading to significant motor disability [1, 2]. All patients become wheelchair users after the age of 15 years and experience progressive joint contractures and spinal deformities. Dysarthria develops in the third decade of life and exacerbate the acoustic startle response [2]. A normal nerve conduction study seems to rule out this condition. No mental impairments have been identified [3].

More than 70 cases have been recorded, two in Egypt and all others in Brazil [4]. This syndrome results from homozygous deletions located in the noncoding upstream region of the KLC2 gene on chromosome 11q13 (chr11.hg19: g.66,024,557_66,024,773del), which upregulates the expression of the Kinesin Light Chain 2 (KLC2) gene. Kinesins are protein motors involved in moving vesicles along microtubules, which is crucial in axonal transport [4]. Patients also have low vision secondary to non-progressive congenital optic atrophy [2, 5]. Apart from optic atrophy and fixation nystagmus, no other information is available about the ocular manifestations of this disease.

To the best of our knowledge, this is the first case of SPOAN syndrome with retinopathy.

## Case presentation

A two-year-old boy with a complaint of eye poking and a lack of eye contact was referred to our clinic. He was the first child of consanguineous healthy Caucasian parents. He was born on week 42 of an uncomplicated pregnancy with a normal birth weight (3200 grams), length, and head circumference.

A physical examination disclosed mild scoliosis and limited knee extension. A neurological examination demonstrated spastic paraplegia with severe developmental delay. He had previously been diagnosed with SPOAN syndrome by a neuro-pediatrician after ruling out other causes of hereditary spastic paraplegia (HSP) as well as TORCH group congenital infections. Genetic testing supported diagnosis of SPOAN syndrome by detecting a likely pathogenic novel variant in KLC2 gene. After the confirming diagnosis of SPOAN syndrome, he was referred to our clinic for an evaluation of visual inattention.

**Fig. 1:**
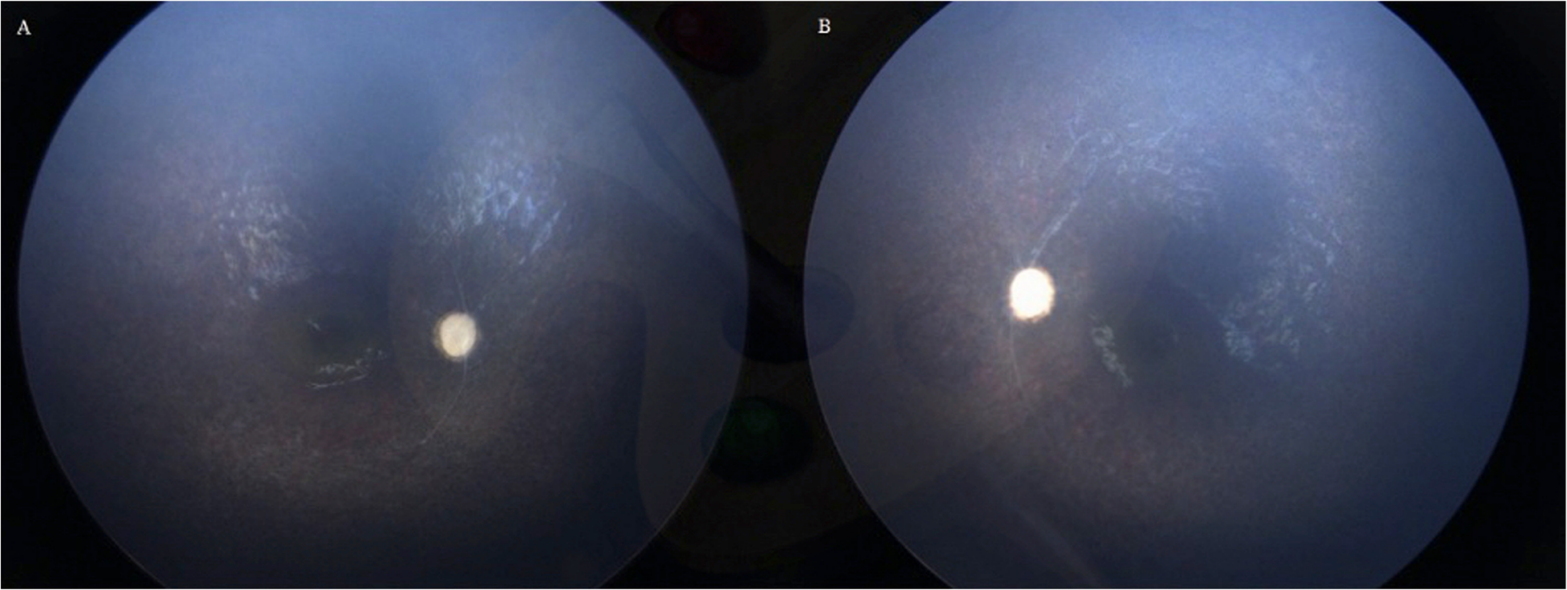
2-year-old child with SPOAN syndrome. Fundus photographs (**a** and **b**; RetCam: Clarity Medical Systems, Pleasanton, CA) showed diffuse pigment mottling from parafoveal toward the peripheral retina, severe vascular attenuation, and optic atrophy. D)

A complete ophthalmic examination (including cyclorefraction, eye motility, and alignment examination, slit-lamp examination, and indirect ophthalmoscopy) was performed. The cyclorefraction of each eye was + 6.00 D. The anterior segment was unremarkable. The patient displayed an oculo-digital reflex (eye poking) and no visual attention without any ptosis, nystagmus, or restricted eye movements. Fundus examination revealed optic disc atrophy, diffuse pigment mottling, severe arterial and venous narrowing, occluded vessels, and completely non-vascularized peripheral retina in both eyes (Figs. 1A and B). Considering that there was no previous report of retinopathy in SPOAN syndrome or previous presence of oculo-digital reflex, high hyperopia, fundus dramatic appearance, or consanguinity of the patient’s parents, a full-field ERG (Metrovision, Perenchies, France) and additional genetic testing were conducted to rule out Leber’s congenital amaurosis (LCA).

**Fig. 2:**
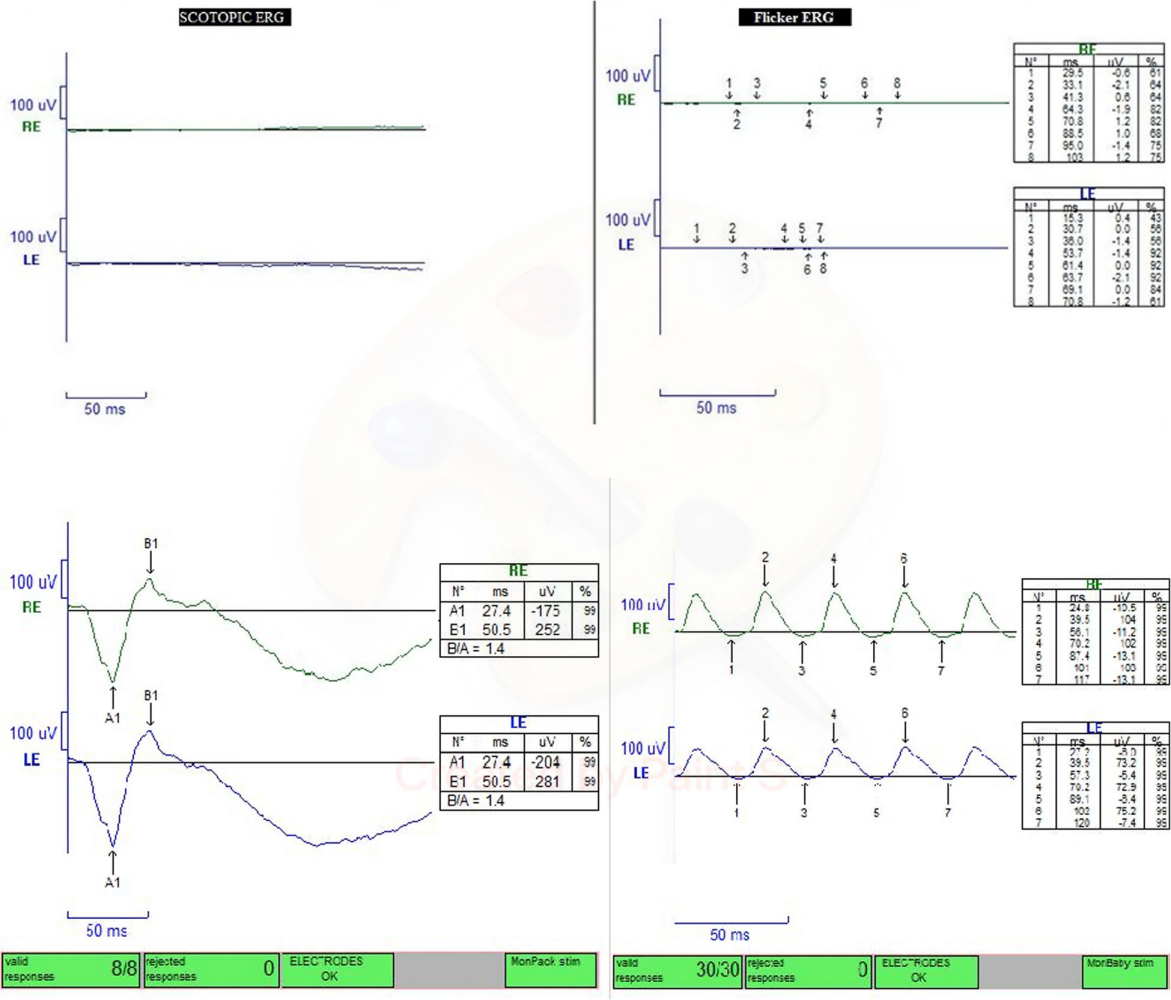
Electroretinography (ERG) under light general anesthesia showed a flat response in both scotopic and photopic ERG (Top row: patient’s ERG, bottom row: normal ERG )

Full-field ERG (10 scotopic and flicker) was performed according to the protocol of the International Society for Clinical Electrophysiology of Vision (ISCEV) [6] with fully dilated pupils (6 mm). Recording electrodes were scleral contact lenses, and flat scotopic and flicker responses were recorded (Fig. 2).

Based on these results, we decided that severe pigmentary retinopathy in our patient, although not reported previously, can be attributed to SPOAN syndrome.

Unfortunately, a few months later, we were informed during a telephone follow-up that after an episode of febrile disease—accompanied by pleural effusion, a decreased level of consciousness, and possible seizures—the child died.

## Genetic analysis

At first step of genetic investigations, patient’s blood sample were collected in EDTA containing tubes. Genomic DNA was extracted from whole blood. A solo whole exome sequencing was performed on patient DNA to investigate causative genomic variant.

Variant calling and filtering was done using Genome Analysis toolkit (GATK-v3.4.0) and detected variants were annotated. Proper filtering and then interpretation of a short list of variants in terms of pathogenicity was performed based on ACMG (American College of Medical Genetics and Genomics) guideline for variant interpretation.

Above investigation resulted in detection of a homozygous single nucleotide variant on chromosome 11 position 66,033,625 (based on hg19 genome assembly). This missense variant is located in exon 14 of *KCL2* gene (NM_022822.3: c.1664G > A; p.Arg555His).

The detected variant was not reported in human mutation databases. The variant is absent in population databases (ExAC, 1000G, and our local database). Multiple lines of *in silico* computational analysis (Mutation Taster, CADD, SIFT, DANN, etc.) support the deleterious effect of this variant on the gene or gene product(s). Conservation assessments tools support conserved nature of this variant (PhyloP is 4.65 for vertebrates, PhastCons is 1 for vertebrates and mammalians).

Analysis of variant in patient’s parents confirmed their heterozygosity for this variant. In addition, patient’s phenotype was consistent with *KLC2*-associated phenotypes. Having such information and based on ACMG guideline, this variant was classified as a likely pathogenic variant and diagnosis of patient’s phenotype was confirmed.

It is worth to mention that focus on genes associated to close ophthalmologic phenotypes such as Leber congenital amaurosis (LCA) did not detect any variant with strong pathogenic scores. Having this another missense variant was detected in ***FXDR*** gene (NM_001258013:c.C556T:p.R186W). This variant has a very low frequency in population databases with only 4 heterozygous in gnomAD exomes and genomes database. This variant was reported recently as a likely pathogenic variant in Clinvar.

Multiple lines of *in silico* computational analysis (Mutation Taster, CADD, SIFT, DANN, etc.) support the deleterious effect of this variant on the gene or gene product(s). Conservation assessments tools support conserved nature of this variant (PhyloP is 2.2669 for vertebrates, PhastCons is 1 for vertebrates). Having these feature and based on ACMG guideline, this variant was classified as a likely pathogenic variant. Considering patient phenotype, we cannot rule out possibility of double diagnosis in this patient.

## Discussion and conclusion

SPOAN is a complicated form of HSP that leads to an early and severe handicap. The motor and functional problems of these patients have been studied extensively [1, 2, 5]. Still, there is no information about the ocular manifestation of this syndrome other than the non-progressive optic atrophy and fixation nystagmus [5].

Symptoms related to optic atrophy such as low vision and fixation nystagmus manifest early in life and are typically not progressive [5]. Macedo-Souza et al. evaluated 61 individuals diagnosed with SPOAN and found optic atrophy in all individuals except in three older patients with cataracts who reported a previous history of poor vision. Fixation nystagmus was observed in 46 cases and was absent in 15 others [5]. Life expectancy does not seem to be decreased by this syndrome [2]. Also, ERG was performed by Macedo-Souza et al. in 3 patients, all with normal result [1, 5] while ERG in our patient showed a flat response.

This study is the first to report SPOAN syndrome with retinopathy, which is characterized by extreme vascular narrowing, diffuse retinal pigment mottling, and avascular peripheral retina. Congenital TORCH infections can be associated with pigmentary retinopathy. However, a systemic workup for TORCH infections was negative in our patient, and, notably, a flat ERG is unusual in TORCH. Using whole-exome sequencing, we also evaluated the patient for LCA that can present with similar ocular findings, which were negative. It seems that severe pigmentary retinopathy in our patient can be attributed to homozygosity for a mutation in KLC2 genes, which is associated with SPOAN syndrome.

Another remarkable subject in our patient was the low age of presentation. The neurological examination revealed a significant developmental delay and paraplegia. He was unable to sit alone at the age of two, while Galvão et al. reported that the mean age at loss of ambulation was 10.78 ± 5.5 years [2]. In our case, ophthalmic manifestations seem to present themselves at a younger age and with more severity than what has been previously reported. This difference might be explained by differences in genetic mutations and resultant disease severity [4]. Some unprecedented aspects of this case may be related to the novelty of the mutation (KLC2: p.R555H: c. G1664A). Therefore, there may be a wide spectrum of clinical features, age of presentation, and severity of pathologic findings in SPOAN syndrome.

Another hypothesis that might explain the atypical features in this patient is the possible synergism between two homozygous variants found in KLC2 and FDXR genes. Even though the variant found in FDXR gene was classified as a variant of unknown significance, it is intriguing that variants in this gene have previously been associated with optic atrophy.

Prior to this report, SPOAN syndrome has been reported only in Brazilian and Egyptian patients; our patient was the first reported Iranian patient with this syndrome. All previous cases of SPOAN syndrome had a common microdeletion in non-coding region of KLC2 gene. The missense variant found in our patient is the first single nucleotide variant reported as the cause of SPOAN syndrome. Differences in presentation of our patient with previously reported cases might be related to the allelic heterogeneity, although the effect of ethnic difference on variation on clinical presentation cannot be underestimated.

In conclusion, we reported a novel mutation in KLC2 gene associated with SPOAN syndrome and also described pigmentary retinopathy in this syndrome for the first time. Therefore, we recommend a full retinal imaging evaluation is necessary for the previous reported cases of SPOAN syndrome to better understand the etiology of the visual impairment.

## Data Availability

Not applicable. This is a case report.

## Abbreviations

SPOAN syndrome: Spastic paraplegia, optic atrophy, neuropathy
ERG: Electroretinogram
KLC2: Kinesin Light Chain 2
HSP: Hereditary spastic paraplegia
LCA: Leber’s congenital amaurosis
ISCEV: International Society for Clinical Electrophysiology of Vision

## References

[1] Macedo-Souza LI, Kok F, Santos S, Amorim SC, Starling A, Nishimura A (2005). Spastic paraplegia, optic atrophy, and neuropathy is linked to chromosome 11q13. Ann Neurol.

[2] Galvao CRC, Cavalcante PMA, Olinda R, Graciani Z, Zatz M, Kok F (2019). Motor impairment in a rare form of spastic paraplegia (Spoan syndrome): a 10-year follow-up. BMC Neurol.

[3] Amorim S, Heise CO, Santos S, Macedo-Souza LI, Zatz M, Kok F (2014). Nerve conduction studies in spastic paraplegia, optic atrophy, and neuropathy (SPOAN) syndrome. Muscle Nerve.

[4] Melo US, Macedo-Souza LI, Figueiredo T, Muotri AR, Gleeson JG, Coux G (2015). Overexpression of KLC2 due to a homozygous deletion in the non-coding region causes SPOAN syndrome. Hum Mol Genet.

[5] Macedo-Souza LI, Kok F, Santos S, Licinio L, Lezirovitz K, Cavacana N (2009). Spastic paraplegia, optic atrophy, and neuropathy: new observations, locus refinement, and exclusion of candidate genes. Ann Hum Genet.

[6] Constable PA, Bach M, Frishman LJ, Jeffrey BG, Robson AG (2017). International Society for Clinical Electrophysiology of V. ISCEV Standard for clinical electro-oculography (2017 update). Doc Ophthalmol.

